# Cardiometabolic-related dietary patterns and thyroid function: a population-based cross-sectional study

**DOI:** 10.1186/s40001-023-01553-1

**Published:** 2023-12-18

**Authors:** Nazanin Moslehi, Saba Mohammadpour, Parvin Mirmiran, Ladan Mehran, Fereidoun Azizi

**Affiliations:** 1 Nutrition and Endocrine Research Center, Research Institute for Endocrine Sciences, Shahid Beheshti University of Medical Sciences, No. 24, Shahid Arabi St, Yemen Blvd, Chamran Exp, Tehran, 1985717413 Iran; 2 Department of Clinical Nutrition and Dietetics, Faculty of Nutrition and Food Technology, National Nutrition and Food Technology Research Institute, Shahid Beheshti University of Medical Sciences, No. 7, Shahid Hafezi St., Farahzadi Blvd., Shahrak-e-qods, Tehran, 1981619573 Iran; 3 Endocrine Research Center, Research Institute for Endocrine Sciences, Shahid Beheshti University of Medical Sciences, Tehran, Iran

**Keywords:** Hypothyroidism, Hyperthyroidism, TPOAb, Euthyroidism, Thyroid-stimulating hormone, Thyroxine, Nutrition

## Abstract

**Background:**

Little is known about the association of dietary patterns with thyroid function. Since thyroid function and cardiometabolic variables are inter-related, we investigated whether cardiometabolic-related dietary patterns are associated with thyroid function.

**Methods:**

This cross-sectional study included 3520 Tehran Lipid and Glucose Study participants. Reduced rank regression was used to find dietary patterns with body mass index, serum fasting glucose, triglycerides, HDL-C, and systolic and diastolic blood pressures as response variables. Two patterns were retained, one based on 35 food groups (native-based pattern) and the other based on the European Prospective Investigation into Cancer and Nutrition Germany (EPIC) food grouping (*n* = 33). A confirmatory cardio-metabolic dietary pattern was also created according to the weight of food groups proposed by the Framingham Offspring Study (FOS). The association of each pattern with thyroid-stimulating hormone (TSH), free thyroxine, and thyroid peroxidase antibody (TPOAb) and the odds of thyroid dysfunction was examined by linear and logistic regression, respectively.

**Results:**

The two exploratory dietary patterns were highly correlated and associated with greater TSH levels in euthyroid participants. The adjusted odds ratio (95% CI) of subclinical hypothyroidism per one standard deviation was 1.14 (1.01, 1.28) for the native-based pattern and 1.16 (1.03, 1.31) for the EPIC-based pattern. The odds of subclinical hypothyroidism was significantly greater in the second and third tertiles of the native-based pattern compared to the first tertile in the adjusted model (p-trend = 0.005). The odds of subclinical hypothyroidism increased across the tertiles of the EPIC-based pattern, but the odds was significantly higher only in tertile 3 compared to tertile 1, with an OR (95% CI) of 1.44 (1.07, 1.94) in the adjusted model. The adjusted odds of clinical hypothyroidism were greater in tertile 3 of the native-based pattern compared with tertile 1 (OR = 1.65, 95% CI 1.04, 2.62). The patterns were unrelated to hyperthyroidism or TPOAb positivity. The FOS-based confirmatory score was unrelated to thyroid function.

**Conclusions:**

A diet high in fast foods, soft drinks, and legumes and low in confectionery, potatoes, butter, and jam and honey was associated with higher TSH levels in euthyroidism and higher odds of subclinical hypothyroidism.

**Supplementary Information:**

The online version contains supplementary material available at 10.1186/s40001-023-01553-1.

## Introduction

The principal hormones released by the thyroid gland, thyroxine (T4) and triiodothyronine (T3) play a crucial role in various physiological processes within the body [1]. These hormones are essential for the normal functioning of the cardiovascular, metabolic, nervous, musculoskeletal, and reproductive systems. Therefore, both subclinical and clinical thyroid dysfunction have detrimental effects on numerous aspects of health [2–6].

Genetic factors, sex, age, stress, lifestyle factors, pollutants, and diet have been demonstrated to affect thyroid function [7, 8]. It is well-known that adequate dietary iodine and selenium intakes are necessary for normal thyroid function. Moreover, there are numerous indications that other food parameters, such as vitamin D, zinc, iron, copper, soy, brassica vegetables, seaweed, and ultra-processed foods, affect thyroid function; however, insufficient research has been conducted on these issues to date [8]. A few studies are also available on the associations of a certain dietary pattern with thyroid-stimulating hormone (TSH) and thyroid hormones [9–11]. In a cross-sectional study involving Italian participants, a higher score for the Mediterranean diet was associated with reduced levels of free T3 (FT3) and free T4 (FT4) but not TSH [9]. A cross-sectional study using the National Health and Nutrition Examination Survey (NHANES) also found a positive association between the dietary inflammatory index score and total T4 in males [10]. In addition, a cross-sectional study of healthy Croatian adults identified multiple dietary factors with distinct characteristics that were associated with FT3, FT4, and TSH. These factors were identified using principal component analysis (PCA) [11].

PCA identifies patterns of commonly consumed foods by grouping food items or food groups based on their correlation with each other. PCA discovers linear combinations (known as principal components) of food items or food groups that capture the most significant variations in individuals’ dietary choices. Unlike PCA, the reduced rank regression (RRR) does not prioritize explaining the variance between foods. Instead, RRR aims to identify linear functions of predictors (such as food groups) that can explain the maximum amount of variation in a set of intermediate response variables (such as biomarkers) [12–14]. Therefore, the dietary pattern that RRR has maintained may better reflect the significance of the dietary pattern in relation to a condition or disease.

Except for the study by Brdar et al. [11] that investigated the association of dietary patterns derived from PCA with TSH and thyroid hormones, no other study has examined the association of dietary patterns with thyroid function. Therefore, the aim of the present study was to identify dietary patterns related to the six cardio-metabolic variables (body mass index (BMI), fasting serum concentrations of glucose, triglycerides, HDL-C, systolic blood pressure (SBP), and diastolic blood pressure (DBP) in our study population and then to investigate the association of the dietary patterns with TSH and thyroid hormones in euthyroid participants and the odds of thyroid dysfunction. We also examined whether a cardiometabolic-related dietary pattern suggested by the Framingham Offspring Study (FOS) [15] was associated with cardio-metabolic variables and thyroid function in our study population.

## Methods

### Participants

**Fig. 1:**
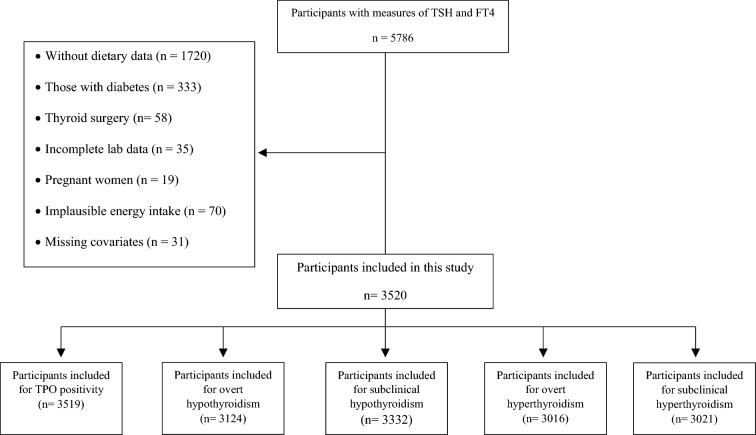
Selection of the study participants

The present investigation is a cross-sectional study that employs data obtained from the Tehran Lipid and Glucose Study (TLGS). The TLGS is prospective, population-based research conducted with a representative sample of participants recruited from district 13 of Tehran, Iran [16]. The thyroid function of a sample of 5786 individuals, randomly selected from a total of 10368 participants aged ≥ 20 years, was evaluated at baseline (1999–2001), as well as during the third examination (2005–2008) and fourth examination (2008–2011) follow-up periods [17]. Dietary intake data has been collected since the third follow-up assessment. For the present study, participants without dietary data (*n* = 1720), those with diabetes (*n* = 333), those who had undergone thyroid surgery (*n* = 58), those with incomplete laboratory data (*n*  = 35), and pregnant women (*n* = 19) were excluded. After further exclusion of participants with implausible energy intakes (*n*  = 70) and those with missing covariates (*n*  = 31), the study included 3,520 participants with valid thyroid function tests and dietary data. For analysis of the associations of dietary patterns with TSH, FT4, and thyroid peroxidase antibody (TPOAb), 2991 participants with euthyroidism were included. Figure 1 provides the number of participants included in each thyroid dysfunction. Implausible energy intake was determined based on sex-specific percentiles 1 and 99.

This investigation was carried out in accordance with the principles outlined in the Declaration of Helsinki. The institutional review committees at the Research Institute for Endocrine Sciences, Shahid Beheshti University of Medical Sciences approved the study's design. Prior to their participation, all individuals involved in the study provided informed consent.

### Dietary assessment

Dietary data were gathered using a valid and reliable semi-quantitative food frequency questionnaire (FFQ) [18, 19]. By conducting individual in-person interviews with each participant, information on the quantity and frequency of 168 food items ingested by the participants in the previous year was gathered. The daily intake of each food item was estimated based on its consumption quantity and frequency. The classification of food items was conducted by grouping them into 35 distinct categories, taking into account both nutrient similarities and the dietary habits of the Iranian population (Additional file 1: Table S1). In addition, the dietary items were categorized into 33 food groups based on the European Prospective Investigation of Cancer and Nutrition in Germany (EPIC) study (Additional file 1: Table S2) [15].

### Demographic and clinical assessments

A questionnaire was used to assess the demographic characteristics, medical history, and medications of the participants. Participants were categorized as never, current, and former for smoking status, as < 6, 6–12, ≥ 12 years for educational status, and as married and others (including single, divorced, and widowed) for marital status. Furthermore, the study population was stratified into two groups based on their employment status (full-time job occupation: yes or no) and family history of diabetes (yes or no).

Weight and height were measured with the individual barefoot, standing, and wearing light clothing. BMI was calculated as the ratio of weight (kg) to height (m^2^). The waist circumference was measured at the narrowest position between the iliac crest and the lowest rib. On the right arm, SBP and DBP were measured twice with a 15-min interval using a standard mercury sphygmomanometer (Riester, Jungingen, Germany); the average of the two measurements was used.

### Biochemical and thyroid function assessments

Blood glucose, triglycerides, and HDL-C concentrations were measured on fasting serum samples on the day of the blood draw using the enzymatic calorimetric method with Pars Azmun commercial kits (Pars Azmun commercial kits, Tehran, Iran). Intra- and inter-coefficients of variations (CV) were 2.2% for serum glucose, 0.6 and 1.6% for triglycerides, and 0.5 and 2% for HDL-C [20]. The electrochemiluminescence immunoassay method with Roche Diagnostic’s kits was utilized to measure FT4 and TSH in serum samples that were stored at – 70 °C. The intra- and inter-CV for FT4 and TSH, respectively, were 1.3% and 3.7%, and 1.5% and 4.5%. The measurement of TPOAb was conducted using an immunoenzymometric assay kit (IEMA; Monobind, Costa Mesa, CA) and the Sunrise ELISA reader (Tecan Co., Salzburg, Austria). The intra- and inter-CVs were determined to be 3.9% and 4.7%, respectively [21].

The reference ranges of 0.32–5.06 mU/L for TSH and 0.91–1.5 ng/dL for FT4 that were established for the study population [22] were used to classify participants according to their thyroid function. Those whose TSH levels were within the reference range without the use of thyroid or antithyroid drugs were categorized as euthyroid. Clinical hypothyroidism was diagnosed as TSH > 5.06 mU/L and FT4 < 0.91 ng/dL or receiving levothyroxine. Subclinical hypothyroidism was defined as TSH > 5.06 mU/L with normal FT4. Clinical hyperthyroidism was defined as TSH < 0.32 mU/L with FT4 > 1.55 ng/dL or taking anti-thyroid medications. Subclinical hyperthyroidism was defined as TSH < 0.32 mU/L with normal FT4. The serum TPOAb levels of ≥ 35 IU/ml in women and ≥ 32 IU/ml in men were defined as TPOAb positivity [21].

### Statistical analysis

Characteristics of the participants based on their thyroid function status were compared using Analysis of Variance (ANOVA) for normal-distributed variables, the Kruskal–Wallis test for non-normal-distributed variables, and the Chi-squared test for categorical variables.

To identify dietary patterns related to the predefined cardiometabolic variables of BMI, fasting serum concentrations of glucose, triglycerides, and HDL-C, and SBP and DBP, two exploratory dietary patterns were created in SAS (version 9.1.3) using the RRR method of PROC PLS. One pattern is based on our study food grouping (35 food categories; the native-based pattern), whereas the other is based on the EPIC research food grouping (33 food groups; the EPIC-based pattern). Food groups were utilized as predictor variables after being adjusted for age, sex, and energy consumption using the residual model [23]. The response variables were BMI, fasting serum concentrations of glucose, triglycerides, and HDL-C, and SBP and DBP. Because of the non-normal distribution of triglycerides, the Ln-transformation was utilized. The first pattern was retained and used in the statistical analyses.

A confirmatory dietary pattern score was also calculated based on a predefined RRR_dietary pattern proposed by the FOS with the aforementioned six cardio-metabolic biomarkers as response variables and the EPIC food grouping (FOS-based pattern) [15]. The residual adjusted values of the 33 food groups were converted to z scores; the z score values were multiplied by factor loadings reported by the FOS, and the resulting values were added [24]. The factor loadings of the FOS dietary pattern are reported in Additional file 1: Table 2.

Pearson correlation was used to determine the correlation between food groups with factor loadings ≥ 0.20, dietary pattern scores, and response variables. Using linear regression analysis, associations between dietary pattern scores and TSH, FT4, and TPOAb in euthyroid participants were investigated. Results were expressed as unstandardized β [95% confidence interval (CI)] per standard deviation (SD) of dietary scores. Logistic regression was used to investigate the odds of thyroid dysfunction, such as clinical and subclinical hypothyroidism, clinical and subclinical hyperthyroidism, and TPOAb positivity, across the tertiles of dietary patterns, considering the first tertile as the reference group. The P for trend across tertiles was calculated by considering the tertiles' median values as the continuous variable. The odds of each thyroid dysfunction was also estimated per SD of the dietary patterns. Results were reported as odds ratios (OR) and 95% CIs. Based on the literature, age, sex, smoking, marital status, education, occupation, family history of diabetes, and TPOAB positivity (except when it was an outcome) were considered potential confounders. The univariate association of these confounding variables with each dependent variable was evaluated, and they were included in the statistical analysis if the *p* value for the association with the dependent variables was ≤ 0.2. The statistical analysis was conducted using the SPSS software (version 20; IBM Corp., Armonk, NY, USA), and statistical significance was set at p ≤ 0.05.

## Results

The mean ± SD age of 3520 participants included in this study was 42.9 ± 12.3 years, and 57.2% were women. Of the participants, 133 had clinical hypothyroidism, 341 had subclinical hypothyroidism, 25 had clinical hyperthyroidism, and 30 had subclinical hyperthyroidism. The characteristics of the participants according to thyroid function state are presented in Table 1. Participants with clinical hypothyroidism and hyperthyroidism were significantly older than those with normal thyroid function. Those with subclinical hypothyroidism had significantly lower fasting serum glucose, whereas those with subclinical hyperthyroidism had significantly higher fasting serum glucose than those with normal function. The prevalence of smokers and full-time employment was highest in those with subclinical hyperthyroidism. TPOAb positivity was most prevalent in clinical hypothyroidism, followed by clinical hyperthyroidism.

**Table 1:** Characteristics of the participants based on thyroid function^a^

Characteristics	Euthyroidism	Clinical hypothyroidism	Clinical hyperthyroidism	Subclinical hypothyroidism	Subclinical hyperthyroidism	*p* value
Number	2991	133	25	341	30	
Age, years	42.8 ± 12.4	45.5 ± 11.2	49.1 ± 18.2	41.8 ± 11.5	42.8 ± 10.9	0.006 ^b^
Women, %	53.6	82.7	60	79.5	46.7	< 0.001^c^
Smoking status, %						< 0.001 ^c^
* Never*	78.1	87.2	76	89.7	56.7	
* Current*	12.4	7.5	8	5.3	30	
* Former*	9.5	5.3	16	5	13.3	
Education, %						0.028 ^c^
< * 6 years*	15.8	17.3	32	18.5	20	
* 6–12 years*	58.8	66.9	52	58.9	66.7	
* ≥ 12 years*	26.2	15.8	16	22.6	13.3	
Marital status						0.184 ^c^
* Married*	83.9	88	72	84.2	93.3	
* Others*	16.1	12	28	15.8	6.7	
Full-time job occupation, %	47.9	28.6	32	25.2	50	< 0.001 ^c^
Family history of type 2 diabetes, %	37.5	34.6	28	35.5	43.3	0.680 ^c^
BMI, kg/m^2^	27.5 ± 4.58	29.2 ± 5.16	27.7 ± 5.47	28.2 ± 4.84	26.8 ± 3.93	< 0.001 ^b^
HDL-C, mg/dL	45.3 ± 10.9	47.8 ± 12.4	45.4 ± 11.7	47.6 ± 11.9	47.6 ± 9.84	0.004 ^b^
WC, cm	92.4 ± 11.8	94.1 ± 12.6	94.3 ± 14.12	91.70 ± 12.1	92.4 ± 10.5	0.349 ^b^
FSG, mg/dL	92.0 ± 9.50	91.9 ± 10.4	94.0 ± 8.52	90.5 ± 8.80	96.3 ± 9.67	0.004 ^b^
Triglycerides, mg/dL	122 (85, 174)	129 (94, 182)	113 (79, 159)	124 (88.5, 176)	117(89.3, 151)	0.498 ^d^
SBP, mmHg	113 ± 16.2	111 ± 16.2	120 ± 18.9	112 ± 15.7	117 ± 18.3	0.055 ^b^
DBP, mmHg	75.5 ± 10.6	75.0 ± 12.0	74.6 ± 13.1	74.8 ± 10.70	79.0 ± 10.2	0.274 ^b^
TSH, mU/L	2.16 ± 1.08	30.8 ± 81.9	0.38 ± 0.96	6.30 ± 4.69	0.18 ± 0.11	< 0.001 ^b^
FT4, ng/dL	1.20 ± 0.17	0.93 ± 0.38	1.94 ± 0.68	1.18 ± 2.23	1.26 ± 0.17	< 0.001 ^b^
TPOAb, U/L	5.19 (3.13, 10.4)	164 (13.3, 446)	8.67 (3.20, 103)	16.1 (4.92, 141)	6.59 (3.50, 11.6)	< 0.001 ^d^
TPOAb positivity, %	10.3	69.2	48	40.5	13.3	< 0.001 ^c^

*BMI* body mass index; *DBP* diastolic blood pressure; *FT4* free thyroxine; *FSG* fasting serum glucose; *HDL-C* high-density lipoprotein cholesterol; *SBP* Systolic blood pressure; *TSH* thyroid-stimulating hormone; *TPOAb*Thyroid peroxidase antibody; *WC* Waist circumference

^a^Data are reported as mean ± standard deviation for normally distributed variables, median (interquartile range) for non-normally distributed variables, and percentage for categorical variables

^b^Based on ANOVA

^c^Based on Chi-square test

^d^Based on the Kruskal–Wallis test

Both exploratory dietary patterns accounted for 4% of the food group variance. The native-based pattern explained 2.1% of the variance in the total response variables, while the EPIC-based pattern explained 1.9%. The native-based pattern was highly (factor loading ≥ 0.2) positively loaded with fast foods, pickled vegetables, breads, soft drinks, legumes, and cooked vegetables but negatively loaded with dried fruits, confectionary, potatoes, butter, and jam and honey. The EPIC-based pattern was highly positively associated with pizza, soft drinks, legumes, and processed meats and negatively associated with fried potatoes, confectionery, cooked potatoes, jam and honey, and cake and cookies. The factor loadings of each food group for the native-based pattern are described in Additional file 1: Table S1, while those for the EPIC-based pattern are described in Additional file 1: Table S2. Both patterns were positively correlated with all response variables, with the highest and lowest correlations with fasting serum glucose and HDL-C, respectively. The FOS-based pattern only showed a weak positive association with BMI, SBP, and DBP **(**Table 2**)**.

**Table 2:** Factor loadings and correlation coefficient between the dietary pattern scores, food groups, and response variables ^a,b, c^

Food groups (g/d)	Factor loadings	Body mass index	Fasting blood glucose	Ln-triglycerides	HDL-C	Systolic blood pressure	Diastolic blood pressure
Native-based exploratory pattern ^d^							
Direct association							
Fast foods	0.36	0.06 (0.001)	0.12 (< 0.001)	0.02 (0.199)	0.04 (0.030)	0.03 (0.095)	0.05 (0.001)
Pickled vegetables	0.30	0.03 (0.126)	0.11 (0.002)	0.02 (0.193)	0.05 (0.002)	0.03 (0.095)	0.05 (0.001)
Breads	0.26	0.06 (0.001)	0.06 (0.001)	0.07 (< 0.001)	− 0.001 (0.939)	0.03 (0.136)	0.05 (0.004)
Soft drinks	0.26	0.08 (< 0.001)	0.05 (0.007)	0.06 (< 0.001)	− 0.03 (0.136)	0.03 (0.048)	0.05 (0.005)
Legumes	0.22	− 0.03 (0.227)	0.14 (< 0.001)	− 0.02 (0.227)	0.10 (< 0.001)	0.01 (0.672)	0.03 (0.080)
Cooked vegetables	0.22	0.04 (0.020)	0.045 (0.008)	0.01 (0.568)	0.04 (0.026)	0.04 (0.039)	0.06 (< 0.001)
Inverse association							
Dried fruits	− 0.28	− 0.05 (0.002)	− 0.09 (< 0.001)	− 0.01 (0.417)	− 0.05 (0.005)	− 0.01 (0.491)	− 0.06 (< 0.001)
Confectionary	− 0.27	− 0.07 (< 0.001)	− 0.08 (< 0.001)	− 0.03 (0.103)	− 0.02 (0.268)	− 0.02 (0.168)	− 0.04 (0.02)
Potatoes	− 0.26	− 0.07 (0.001)	− 0.07 (< 0.001)	− 0.01 (0.537)	− 0.03 (0.111)	− 0.02 (0.214)	− 0.06 (0.002)
Butter	− 0.22	− 0.08 (< 0.001)	− 0.02 (0.148)	− 0.04 (0.018)	− 0.01 (0.608)	− 0.04 (0.032)	− 0.03 (0.061)
Jam-honey	− 0.21	− 0.06 (< 0.001)	− 0.05 (0.001)	− 0.05 (0.008)	0.02 (0.190)	− 0.02 (0.206)	− 0.03 (0.084)
Total dietary score	–	0.18 (< 0.001)	0.22 (< 0.001)	0.12 (< 0.001)	0.06 (< 0.001)	0.08 (< 0.001)	0.17 (< 0.001)
EPIC-based exploratory pattern ^d^							
Direct association							
Pizza	0.37	0.03 (0.052)	0.133 (< 0.001)	0.01 (0.610)	0.06 (< 0.001)	0.02 (0.221)	0.06 (0.001)
Soft drink	0.28	0.08 (< 0.001)	0.05 (0.007)	0.06 (< 0.001)	-0.03 (0.136)	0.03 (0.048)	0.05 (0.005)
Legumes	0.25	− 0.03 (0.227)	0.14 (< 0.001)	− 0.02 (0.227)	0.10 (< 0.001)	0.01 (0.672)	0.03 (0.080)
Processed meats	0.20	0.07 (< 0.001)	0.02 (0.185)	0.03 (0.100)	− 0.01 (0.572)	0.03 (0.101)	0.04 (0.011)
Inverse association							
Fried potato	-0.42	− 0.06 (0.001)	− 0.15 (< 0.001)	− 0.02 (0.254)	− 0.10 (< 0.001)	− 0.01 (0.395)	− 0.04 (0.014)
Confectionary	-0.29	− 0.07 (< 0.001)	− 0.08 (< 0.001)	− 0.03 (0.108)	-0.02 (0.266)	-0.02 (0.182)	-0.04 (0.020)
Cooked potato	− 0.24	− 0.07 (< 0.001)	− 0.04 (0.017)	− 0.016 (0.334)	004 (0.797)	− 0.03 (0.097)	− 0.05 (0.002)
Jam− honey	− 0.23	− 0.06 (< 0.001)	− 0.05 (0.001)	− 0.05 (0.008)	0.02 (0.190)	− 0.02 (0.206)	− 0.03 (0.084)
Cake & cookies	− 0.20	− 0.03 (0.085)	− 0.04 (0.009)	− 0.05 (0.002)	0.004 (0.793)	− 0.02 (0.327)	− 0.04 (0.021)
Total dietary score	−	0.17 (< 0.001)	0.21 (< 0.001)	0.11 (< 0.001)	0.06 (0.001)	0.07 (< 0.001)	0.14 (< 0.001)
FOS confirmatory pattern							
Direct association							
Meat	0.26	− 0.02 (0.360)	0.002 (0.915)	− 0.04 (0.031)	0.02 (0.295)	0.01 (0.563)	− 0.003 (0.845)
Margarine	0.24	0.00 (0.995)	− 0.04 (0.012)	− 0.01 (0.782)	− 0.02 (0.171)	0.02 (0.183)	− 0.002 (0.928)
Pizza	0.23	0.03 (0.052)	0.133 (< 0.001)	0.01 (0.610)	0.06 (< 0.001)	0.02 (0.221)	0.06 (0.001)
Fried potatoes	0.20	− 0.06 (0.001)	− 0.15 (< 0.001)	− 0.02 (0.254)	− 0.10 (< 0.001)	− 0.01 (0.395)	− 0.04 (0.014)
Other bread	0.21	0.04 (0.014)	0.014 (0.418)	0.02 (0.179)	− 0.02 (0.383)	0.02 (0.344)	0.02 (0.372)
Processed meat	0.20	0.07 (< 0.001)	0.02 (0.203)	0.03 (0.103)	− 0.01 (0.614)	0.03 (0.099)	0.04 (0.010)
Total dietary score	–	0.04 (0.017)	0.001 (0.942)	0.02 (0.315)	− 0.02 (0.187)	0.05 (0.005)	0.04 (0.024)

^a^Results are presented as Pearson correlation coefficients (*p* value)

^b^Food groups were adjusted for age, sex, and energy intake using residual model

^c^Food groups with absolute factor loading ≥ 0.2 are reported

^d^Exploratory patterns were derived using the reduced rank regression

Table 3 shows associations between dietary patterns, TSH, FT4, and TPOAb in individuals with normal thyroid function. In the unadjusted model, there was a 0.08 mU/L (95% CI 0.04, 0.12) and 0.06 mU/L (95% CI 0.03, 0.11) increase in serum TSH concentration per one SD increase in the scores of the native-based pattern and the EPIC-based pattern, respectively. The associations remained significant after taking into account the covariates. These two patterns showed no significant association with FT4. A marginally significant inverse association was observed between the FOS-based pattern and TSH, which became non-significant in the multivariable adjusted model (*p* value = 0.069).

**Table 3:** Association between dietary pattern scores and thyroid stimulating-hormone, free thyroxine, and thyroid peroxidase antibodies in participants with normal thyroid function (n = 2991)^a, b^

	Native− based exploratory pattern		EPIC− based exploratory pattern		FOS− based confirmatory pattern	
	Β (95% CI)	*p* value	Β (95% CI)	*p* value	Β (95% CI)	*p* value
TSH, mU/L						
Unadjusted	0.08 (0.04, 0.12)	< 0.001	0.06 (0.03, 0.10)	0.001	− 0.04 (− 0.08, 0.00)	0.051
Model 1^c^	0.08 (0.04, 0.12)	< 0.001	0.06 (0.03, 0.10)	0.001	− 0.04 (− 0.08, 0.00)	0.049
Model 2^d^	0.08 (0.05, 0.12)	< 0.001	0.07 (0.03, 0.10)	< 0.001	− 0.03 (− 0.07, 0.00)	0.069
FT4, ng/dL						
Unadjusted	− 0.01 (− 0.01, 0.00)	0.060	− 0.01 (− 0.01, 0.00)	0.055	− 0.002 (− 0.01, 0.01)	0.595
Model 1^c^	− 0.01 (− 0.01, − 0.001)	0.025	− 0.01 (− 0.01, − 0.001)	0.022	− 0.002 (− 0.01, 0.00)	0.516
Model 2^e^	− 0.01 (− 0.01, 0.00)	0.066	− 0.01 (− 0.01, 0.00)	0.070	− 0.001 (− 0.01, 0.00)	0.625
TPOAb, U/L						
Unadjusted	− 0.02 (− 0.06, 0.03)	0.459	− 0.02 (− 0.07, 0.02)	0.298	− 0.01 (− 0.05, 0.04)	0.690
Model 1^f^	− 0.02 (− 0.06, 0.03)	0.487	− 0.02 (− 0.07, 0.02)	0.312	− 0.01 (− 0.05, 0.04)	0.704
Model 2^g^	− 0.02 (− 0.06, 0.03)	0.491	− 0.02 (− 0.07, 0.02)	0.317	− 0.01 (− 0.05, 0.03)	0.669

^a^Exploratory patterns were derived using the reduced rank regression

^b^Estimates were based on one standard deviation for each pattern

^c^Adjusted for age (continuous), and sex

^d^Adjusted for age (continuous), sex, smoking (never, current, former), education (< 6, 6–12, ≥ 12 years), having a full-time job (yes, no), and TPOAb positivity (yes, no)

^e^Adjusted for age, sex, smoking (never, current, former), education (< 6, 6–12, and ≥ 12 years), having a full-time job (yes, no), marital status (married, others), family history of diabetes (yes, no), TPOAb positivity (yes, no)

^f^Adjusted for sex

^g^Adjusted for sex, energy intake (continuous), smoking (never, current, former), and having a full-time job (yes, no)

Table 4 presents the associations between dietary pattern scores and the odds of clinical and subclinical hypothyroidism. There was an increase in the odds of clinical hypothyroidism across the tertiles of the native-based pattern (p-trend = 0.033) in the multivariable-adjusted model. The odds of clinical hypothyroidism was significantly higher in tertile 3 compared to tertile 1 (HR = 1.65; 95% CI 1.04, 2.62) in the adjusted model 2. The other dietary patterns were not related to clinical hypothyroidism. The risk of subclinical hypothyroidism was significantly greater in the second and third tertiles of the native-based pattern compared to the first tertile, with ORs (95% CIs) of 1.49 (1.11, 1.98) and 1.54 (1.41, 2.09) in the adjusted model 2 (p-trend = 0.005). As a continuous variable, one SD increase in the dietary pattern score 1 was associated with a 14% higher odds of subclinical hypothyroidism after adjusting for covariates (OR = 1.14, 95% CI 1.01, 1.28). In addition, the odds of subclinical hypothyroidism increased across the tertiles of the EPIC-based pattern, but the odds was significantly higher only in tertile 3 compared to tertile 1, with an OR (95% CI) of 1.44 (1.07, 1.94) in the adjusted model 2. One SD increase in the score of the EPIC-based pattern was also related to a 16% higher odds of subclinical hypothyroidism (OR = 1.16, 95% CI 1.03, 1.31) in the adjusted model 2.

**Table 4:** Odds ratio (95% confidence interval) for hypothyroidism according to the dietary pattern scores ^a^

	Tertile 1	Tertile 2	Tertile 3	p-trend	Per SD	*p* value
Clinical hypothyroidism						
Native-based exploratory pattern						
No. of participants	1041	1041	1042		3124	
No. of cases	38	39	56		133	
Unadjusted	1.00	1.03 (0.65, 1.62)	1.50 (0.98, 2.28)	0.056	1.14 (0.95, 1.36)	0.154
Model 1	1.00	1.03 (0.65, 1.63)	1.55 (1.01, 2.37)	0.042	1.14 (0.96, 1.36)	0.142
Model 2	1.00	1.02 (0.62, 1.67)	1.65 (1.04, 2.62)	0.033	1.16 (0.95, 1.40)	0.141
EPIC-based exploratory pattern						
No. of participants	1041	1041	1042		3124	
No. of cases	43	42	46		133	
Unadjusted	1.00	1.02 (0.67, 1.57)	1.07 (0.70, 1.64)	0.749	1.08 (0.91, 1.29)	0.383
Model 1	1.00	1.01 (0.65, 1.56)	1.08 (0.70, 1.66)	0.733	1.09 (0.91, 1.30)	0.365
Model 2	1.00	1.06 (0.66, 1.69)	1.23 (0.77, 1.69)	0.386	1.13 (0.93, 1.37)	0.212
FOS-based confirmatory pattern						
No. of participants	1042	1041	1041		3124	
No. of cases	50	40	43		133	
Unadjusted	1.00	0.79 (0.52, 1.21)	0.86 (0.56, 1.30)	0.435	0.90 (0.75, 1.06)	0.180
Model 1	1.00	0.78 (0.51, 1.19)	0.84 (0.55, 1.27)	0.378	0.90 (0.74, 1.06)	0.179
Model 2	1.00	0.88 (0.55, 1.40)	0.81 (0.51, 1.27)	0.354	0.88 (0.74, 1.07)	0.144
Subclinical hypothyroidism						
Native-based exploratory pattern						
No. of participants	1111	1111	1110		3332	
No. of cases	89	129	123		341	
Unadjusted	1.00	1.51 (1.14, 2.00)	1.43 (1.08, 1.91)	0.015	1.11 (0.99, 1.25)	0.066
Model 1	1.00	1.47 (1.11, 1.97)	1.47 (1.10, 1.96)	0.010	1.12 (1.00, 1.26)	0.061
Model 2	1.00	1.49 (1.11, 1.98)	1.54 (1.41, 2.09)	0.005	1.14 (1.01, 1.28)	0.041
EPIC-based exploratory pattern						
No. of participants	1110	1111	1111		3332	
No. of cases	96	119	126		341	
Unadjusted	1.00	1.27 (0.96, 1.68)	1.35 (1.02, 1.79)	0.036	1.13 (1.01, 1.26)	0.041
Model 1	1.00	1.23 (0.93, 1.64)	1.34 (1.01, 1.78)	0.043	1.13 (1.01, 1.27)	0.037
Model 2	1.00	1.27 (0.94, 1.71)	1.44 (1.07 1.94)	0.015	1.16 (1.03, 1.31)	0.017
FOS-based confirmatory pattern						
No. of participants	1111	1111	1110		3332	
No. of cases	116	115	110		341	
Unadjusted	1.00	0.98 (0.75, 1.29)	0.94 (0.71, 1.23)	0.643	0.99 (0.89, 1.11)	0.871
Model 1	1.00	0.94 (0.71, 1.24)	0.92 (0.70, 1.22)	0.568	0.99 (0.88, 1.11)	0.858
Model 2	1.00	0.96 (0.72, 1.28)	0.92 (0.70, 1.24)	0.592	1.00 (0.88, 1.12)	0.936

Model 1: Adjusted for age (continuous), and sex

Model 2: Adjusted for age (continuous), sex, smoking (never, current, former), education (< 6, 6–12, ≥ 12 years), having a full-time job (yes, no), and TPOAb positivity (yes, no)

^a^Exploratory patterns were derived using the reduced rank regression

**Fig. 2:**
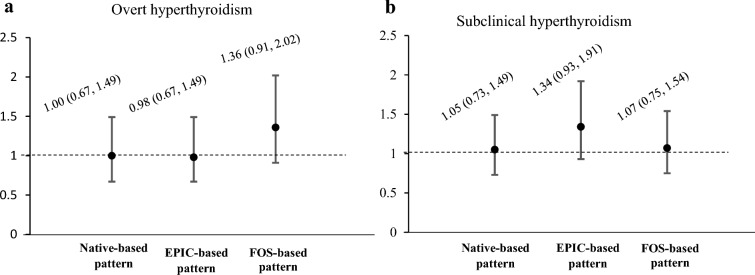
Odds ratio (95% confidence interval) for overt hyperthyroidism (**a**) and subclinical hyperthyroidism (**b**) per one standard deviation in each dietary pattern score. Estimates for overt hyperthyroidism was adjusted for age (continuous), education (< 6, 6–12, ≥ 12 years), having a full-time job (yes, no), marital status (married, others), and TPOAb positivity (yes, no). Estimates for subclinical hyperthyroidism was adjusted for age (continuous), education (< 6, 6–12, ≥ 12 years), marital status (married, others), and smoking (never, current, former). The native and EPIC-based patterns are exploratory patterns retained using reduced rank regression. The FOS-based pattern is a confirmatory cardiometabolic-related pattern computed using the weight reported by the Framingham Offspring Study

Figure 2 illustrates the odds of clinical hyperthyroidism and subclinical hyperthyroidism per SD of each dietary pattern. The dietary pattern was not related to the odds of hyperthyroidism. In addition, no significant association was observed between dietary patterns and the odds of TPOAb positivity (Table 5).

**Table 5:** Odds ratio (95% confidence interval) for TPO positivity according to the dietary pattern scores

	Tertile 1	Tertile 2	Tertile 3	p-trend	Per SD	*p* value
Native-based exploratory pattern						
No. of participants	1174	1172	1173		3519	
No. of cases	178	194	183		555	
Unadjusted	1.00	1.11 (0.89, 1.39)	1.03 (0.83, 1.29)	0.441	0.98 (0.90, 1.08)	0.699
Multivariable ^a^	1.00	1.04(0.83, 1.30)	0.89 (0.71, 1.12)	0.353	0.98 (0.89, 1.07)	0.620
EPIC-based exploratory pattern						
No. of participants	1172	1174	1173		3519	
No. of cases	186	197	172		555	
Unadjusted	1.00	1.07 (0.86, 1.33)	0.91 (0.73, 1.14)	0.867	0.10 (0.91, 1.09)	0.847
Multivariable ^a^	1.00	1.07 (0.86, 1.34)	1.03 (0.82, 1.29)	0.838	0.10 (0.91, 1.09)	0.843
FOS-based confirmatory pattern						
No. of participants	1172	1173	1174		3519	
No. of cases	192	177	186		555	
Unadjusted	1.00	0.91 (0.73, 1.13)	0.96 (0.77, 1.20)	0.728	0.96 (0.88, 1.05)	0.361
Multivariable ^a^	1.00	0.88 (0.71, 1.11)	0.95 (0.76, 1.19)	0.669	0.96 (0.88, 1.05)	0.352

^a^Multivariable model was adjusted for sex, smoking (never, current, former), education (< 6, 6–12, ≥ 12 years), having a full-time job (yes, no), marital status (married, others), and family history of diabetes (yes, no)

## Discussion

We identified two dietary patterns that were positively correlated with all six cardiometabolic markers, including BMI, serum glucose, HDL-C, triglycerides, SBP, and DBP. The native-based pattern was characterized by high intakes of fast foods, pickled vegetables, breads, soft drinks, legumes, and cooked vegetables and low intakes of dried fruits, confectionary, potatoes, butter, and jam–honey. The EPIC-based pattern was characterized by high intakes of pizza, soft drinks, legumes, and processed meats and low intakes of fried potatoes, confectionery, jam and honey, cooked potatoes, butter, cake, and cookies. These two cardiometabolic-related dietary patterns were associated with higher TSH levels in participants with normal thyroid function and an increased odds of subclinical hypothyroidism.

Insufficient studies have examined the relationship between nutrition and thyroid function within the context of dietary patterns [9–11]. Two cross-sectional studies that computed an a priori dietary index indicated a negative association between the Mediterranean diet and FT3 and FT4 [9] and a positive association between the dietary inflammatory index and total T4 [10]. In addition, a cross-sectional study employing the method of PCA identified 18 dietary components with varying associations with thyroid function. High consumption of fruit juices, Cedevita vitamin drinks, and non-alcoholic drinks was associated with lower TSH and higher FT4, whereas high consumption of bacon and sausage was associated with higher FT3 and FT4. A factor with a high positive loading for white bread and a high negative loading for whole grains was also significantly associated with FT4 [11].

In this research, we used two different food groupings, one based on the dietary habits of the study population and the other based on the EPIC food grouping. The two RRR patterns were highly correlated (*r* = 0.85) and both accounted for the same variation in food groups (4%) and response variables (2.1% for the native-based pattern and 1.9% for the EPIC-based pattern). Similar correlations were observed between these patterns and response variables; serum glucose and HDL-C had the highest and lowest correlations, respectively. Nonetheless, the magnitude of the contribution of food to patterns was slightly different, particularly for those food groups that were modified.

Using the RRR method and the EPIC food groups, the FOS also kept a dietary pattern that was linked to the 5 cardio-metabolic variables (BMI, serum glucose, triglycerides, HDL-C, SBP, and DBP). The FOS-suggested dietary pattern was positively associated with BMI, fasting glucose, triglycerides, SBP, and DBP and negatively associated with HDL-C [15]. However, the contribution of food groups to the dietary pattern was different from ours. We also made a simplified dietary pattern based on the FOS factor loadings. However, the FOS-based pattern only showed very weak correlations with SBP and DBP among the cardiometabolic variables, suggesting this pattern was unrelated to cardiometabolic parameters in our population. Using total cholesterol, triglycerides, and HDL-C as intermediate variables and the RRR method, the Whitehall I study suggests two dietary patterns [25]. Moreover, the Multi-Ethnic Study of Atherosclerosis (MESA) identified a dietary pattern that was positively correlated with waist circumference, serum glucose, triglycerides, SBP, and DBP and negatively correlated with HDL-C [26]. The characteristics of diets related to cardio-metabolic variables vary between studies. However, food groups that were consistent in these patterns and ours revealed that a pattern with high intakes of processed meats, pizza, and soft drinks is associated with higher glucose, BMI (or waist circumference), triglycerides, SBP, and DBP. Some prospective studies have also found an association between processed meat and soft drink consumption and metabolic abnormalities such as obesity, elevated blood pressure, and abnormal blood glucose [27, 28].

Cardiometabolic variables and thyroid function are closely and reciprocally associated. Some studies confirmed the causal associations between thyroid function and cardiometabolic health [29–31]. On the other hand, BMI and HDL-C have been suggested to have a causal effect on thyroid function [29, 30, 32]. Moreover, diabetes is also associated with a higher prevalence of thyroid dysfunction [33]. A prospective study also demonstrated a higher incidence of subclinical hypothyroidism among those with metabolic syndrome during an average follow-up period of 4.2 years. Among the components of the metabolic syndrome, high blood pressure and high triglyceridemia showed significant positive associations with subclinical hypothyroidism [34]. The association between these dietary patterns and subclinical hypothyroidism was, therefore, partially explained by cardio-metabolic variables, particularly serum glucose and BMI. Legumes and vegetables were positively loaded into the exploratory dietary patterns identified in the current study. Some plant constituents, such as cyanogenic glucosides, polyphenols, phenolic acids, and alkaloids, have been proposed to have anti-thyroid effects. However, the interference of the constituents with thyroid function depends on their amount of ingestion and iodine status, and the amount consumed through diet is insufficient to affect thyroid function [35].

This study investigates for the first time the association between RRR-retained dietary patterns and thyroid function. The RRR method allows for the generation of hypotheses regarding potential mediators between patterns and thyroid function [14]. Other strengths of the study include the use of population-based data, a relatively large sample size, and a validated dietary assessment instrument. The findings of the current study are clinically important because subclinical hypothyroidism is a common endocrinopathy that can increase the risk of metabolic syndrome, cardiovascular diseases, cognitive decline, and mortality [36–38]. According to this study, dietary patterns may be an important modifiable risk factor for subclinical hypothyroidism.

However, there are also some limitations that should be considered. The cross-sectional design of the study means that causality cannot be established and reverse causation cannot be ruled out. In addition, the study was conducted on the population of Tehran, so the results may not be generalizable to other populations. In addition, a small number of participants had hyperthyroidism. Therefore, the sample size used to examine the association between diet and hyperthyroidism was insufficient. Furthermore, concern for residual or unmeasured confounders remained because of the observational design of the study.

In conclusion, a dietary pattern positively related to cardio-metabolic variables, characterized by high intakes of fast foods (pizza and processed meat), soft drinks, and legumes and low intakes of confectionery, potatoes, butter, and jam and honey, was associated with higher levels of TSH in participants with euthyroidism and an increased odds of subclinical hypothyroidism.

### Supplementary Information

**Additional file 1: Table S1.** Food grouping and food group contributions to the native-based pattern. **Table S2.** Food grouping based on the European Prospective Investigation into Cancer and Nutrition, Potsdam Study and contributions of food groups to the EPIC-based pattern and the FOS-based pattern dietary patterns.

## Data Availability

All the datasets are available from the corresponding author on reasonable request.

## Abbreviations

ANOVA: Analysis of variance
DBP: Diastolic blood pressure
EPIC: European Prospective Investigation of Cancer and Nutrition in Germany
FOS: Framingham Offspring Study
NHANES: National Health and Nutrition Examination Survey
PCA: Principal component analysis
RRR: Reduced rank regression
SBP: Systolic blood pressure
TLGS: Tehran Lipid and Glucose Study
TPOAb: Thyroid peroxidase antibody
TSH: Thyroid-stimulating hormone
T4: Thyroxine
T3: Triiodothyronine
