# Supplementary material for: Cardiometabolic-related dietary patterns and thyroid function: a population-based cross-sectional study
Source: Eur J Med Res. 2023 Dec 18;28:602. doi: 10.1186/s40001-023-01553-1 (PMC10726591; doi:10.1186/s40001-023-01553-1)
Supplement: Supplementary file 1 — Additional file 1: Table S1. Food grouping and food group contributions to the native-based pattern. Table S2. Food grouping based on the European Prospective Investigation into Cancer and Nutrition, Potsdam Study and contributions of food groups to the EPIC-based pattern and the FOS-based pattern dietary patterns. [file 40001_2023_1553_MOESM1_ESM.docx]

**Table S1-** Food grouping and food group contributions to the native-based pattern

| Food groups | Factor loading |
| --- | --- |
| **Breads:** Different types of traditional bread (including lavash, barbari, sanagak, taftun), baguette, bread toast, flour | **0.26** |
| **Pasta-rice:** Pasta, rice, different types of noodles | -0.04 |
| **Potatoes:** Potatoes, French-fries | **-0.26** |
| **Grains:** Rye, bulgur | 0.11 |
| **Biscuits:** Biscuits, Cracker | -0.16 |
| **Cakes:** Yazdi cakes, other kind of cakes and pastries, sweet piroshki | -0.19 |
| **Legumes:** Lentils, beans, chickpeas, soybeans, mung beans, lima beans | **0.22** |
| **Red meat:** Beef, lamb, ground meat, hamburger | -0.02 |
| **Poultry:** Poultry | 0.12 |
| **Fish:** Fish, tuna-fish | -0.02 |
| **Fast foods:** Processed meat, pizza | **0.36** |
| **Organ meat:** Organ meats of chicken, lamb and beef | -0.02 |
| **Eggs:** Eggs | -0.06 |
| **Fermented Dairy:** Yogurt, cheese, dough, kashk | -0.10 |
| **Non-fermented dairy:** Milk, chocolate milk, ice cream, cream | -0.01 |
| **Butter:** Butter | **-0.22** |
| **Olive oil:** Olive, olive oil | -0.03 |
| **Animal fat except butter:** Animal fat | -0.001 |
| **Vegetable fat:** Margarine, hydrogenated vegetable oil | -0.12 |
| **Vegetable oil:** Vegetable oil | 0.11 |
| **Fruits:** Fresh fruits | 0.07 |
| **Dried fruits:** Dried figs, raisin, dried Mulberry, dried Peach, dried apricot, other dried fruits | **-0.28** |
| **Fruit juice:** Carrot juice, orange juice, apple juice, other fruit juices | 0.10 |
| **Canned fruits:** Canned pineapple, other canned fruit | -0.15 |
| **Cooked vegetables:** Vegetables consumed after frying including mixed leafy vegetables, zucchini, celery, eggplants , onion, spinach | **0.22** |
| **Leafy vegetables:** Green leafy vegetables, lettuce, spinach, Cabbage, cauliflower | 0.11 |
| **Non-leafy vegetables:** Tomatoes, cucumber, carrots, squash, pumpkin, green bean, green peas, bell Pepper, green pepper, onion, garlic, shallot , mushroom, Corn, broad bean | 0.11 |
| **Nuts:** Peanuts, almonds, walnuts, pistachios, hazelnuts, roasted seeds | 0.06 |
| **Confectionary:** Sugar cube, sugar, Gaz, candy, Toffee, Sohan, chocolate, Crème Caramel, Candy, halve, halva-ardeh, noghl, ice cream | **-0.27** |
| **Tea-coffee:** Tea, coffee | -0.04 |
| **Chips, salty snacks:** Cheese Puffs, potato Chips | 0.10 |
| **Pickled vegetables:** Torshi, Shoor, pickled cucumber | **0.30** |
| **Jam-honey:** Jam, honey | **-0.21** |
| **Soft drinks:** Soft drink | **0.26** |
| **Sauce:** Mayonnaise, ketchup | 0.17 |
| Variance explained, % |  |
| Food groups | 4.0 |
| Body mass index | 3.0 |
| Fasting blood glucose | 4.7 |
| HDL-C | 0.3 |
| Ln_triglycerides | 1.5 |
| Systolic blood pressure | 0.6 |
| Diastolic blood pressure | 2.7 |
| All response variables | 2.1 |

**Table S2-** Food grouping based on the European Prospective Investigation into Cancer and Nutrition, Potsdam Study and contributions of food groups to the EPIC-based pattern and the FOS-based pattern dietary patterns

| **Food Groups based on EPIC-Potsdam Study** | **Factor loading** | |
| --- | --- | --- |
|  | **FOS-based confirmatory pattern^a^** | **EPIC-based exploratory pattern ^b^** |
| **Whole-grain bread:** Dark bread | -0.13 | 0.12 |
| **Other bread:** White bread, biscuits, other grains, pancakes, wheat germ | **0.21** | 0.11 |
| **Pasta, rice:** Brown rice, white rice, pasta | -0.04 | -0.05 |
| **Cooked potatoes** | 0.03 | **-0.24** |
| **Fried potatoes** | **0.20** | **-0.42** |
| **Cake, cookies:** Cookies, cakes | -0.01 | **-0.20** |
| **Legumes:** Tofu or soybeans, string beans, peas or lima beans, beans or lentils | -0.03 | **0.25** |
| **Meat:** Beef, lamb, hamburger, Beef, calf | **0.26** | -0.04 |
| **Poultry:** Chicken with or without skin | 0.12 | 0.13 |
| **Fish:** Canned tuna fish, fish | -0.04 | -0.02 |
| **Processed meat:** Processed meats | 0.20 | **0.20** |
| **Pizza** | **0.23** | **0.37** |
| **Eggs** | 0.15 | -0.06 |
| **High-fat dairy:** Whole milk, cream | -0.14 | -0.06 |
| **Low-fat dairy:** Skim or low-fat milk, yogurt | 0.05 | -0.08 |
| **High-fat cheese:** Cream cheese, high fat cheese | 0.06 | -0.001 |
| **Low-fat cheese:** low fat cheese | 0.01 | 0.01 |
| **Butter** | -0.06 | **-0.23** |
| **Margarine** | **0.24** | -0.07 |
| **Vegetable oils:** Oil and vinegar salad dressing | -0.11 | 0.13 |
| **Fresh fruit:** Raisins, prunes, bananas, cantaloupe, watermelon, apples, pears, oranges, grapefruits, strawberries, blueberries, peaches | -0.07 | 0.07 |
| **Fruit juice:** Apple juice, orange juice, other fruits juice | -0.09 | **0.11** |
| **Cooked vegetables:** Tomato juice, broccoli, cabbage or coleslaw, cauliflower, cooked carrots, corn, mixed vegetables, winter squash, summer squash, cooked spinach, kale or chard, celery, beets | -0.02 | 0.14 |
| **Raw vegetables:** Brussels sprouts, raw carrots, raw spinach, lettuce, tomatoes | 0.01 | 0.14 |
| **Garlic** | -0.11 | 0.06 |
| **Nuts:** Peanuts, almonds, walnuts, pistachios, hazelnuts, roasted seeds | -0.08 | 0.07 |
| **Confectionery, ice cream:** Sugar cube, sugar, Gaz, candy, Toffee, Sohan, chocolate, Crème Caramel, Candy, halve, halva-ardeh, noghl, ice cream | 0.06 | **-0.29** |
| **Tea** | -0.11 | -0.04 |
| **Coffee** | -0.10 | -0.10 |
| **Chips, salt sticks:** Potato chips, crackers, popcorn | -0.04 | -0.11 |
| **High-energy soft drink:** Cola with sugar | 0.18 | **0.28** |
| **Sweet bread spreads:** Jams, honey | -0.13 | **-0.23** |
| **Sauce:** Mayonnaise, ketchup | 0.07 | 0.16 |
| **Variance explained, %** |  |  |
| Food groups | NR | 4.0 |
| Body mass index | NR | 2.9 |
| Fasting blood glucose | NR | 4.5 |
| HDL-C | NR | 0.3 |
| Ln_triglycerides | NR | 1.2 |
| Systolic blood pressure | NR | 0.5 |
| Diastolic blood pressure | NR | 1.9 |
| All response variables | NR | 1.9 |

^1^ Factor loadings reported by the Framingham Offspring Study.

^2^ Factor loadings obtained in the present study based on the EPIC food grouping using reduced rank regression.

NR; not reported.
